# Categorization of disaster-related deaths in Minamisoma city after the Fukushima nuclear disaster using clustering analysis

**DOI:** 10.1038/s41598-024-53165-2

**Published:** 2024-02-05

**Authors:** Hiroki Yoshimura, Toyoaki Sawano, Michio Murakami, Yuna Uchi, Moe Kawashima, Kemmei Kitazawa, Saori Nonaka, Naomi Ito, Hiroaki Saito, Toshiki Abe, Nobuaki Moriyama, Mamoru Sakakibara, Kazuko Yagiuchi, Mako Otsuki, Arinobu Hori, Akihiko Ozaki, Chika Yamamoto, Tianchen Zhao, Taiga Uchiyama, Tomoyoshi Oikawa, Shinichi Niwa, Masaharu Tsubokura

**Affiliations:** 1 Department of Radiation Health Management, Fukushima Medical University School of Medicine, Fukushima, Japan; 2 School of Medicine, Hiroshima University, Hiroshima, Japan; 3 Department of Surgery, Jyoban Hospital of Tokiwa Foundation, Iwaki, Japan; 4 Research Center for Community Health, Minamisoma Municipal General Hospital, Fukushima, Japan; 5 Department of Health Risk Communication, Fukushima Medical University School of Medicine, Fukushima, Japan; 6 Present Address: Center for Infectious Disease Education and Research, Osaka University, Suita, Japan; 7 Department of Internal Medicine, Soma Central Hospital, Fukushima, Japan; 8 Department of Public Health, Fukushima Medical University School of Medicine, Fukushima, Japan; 9 Reinstatement Support Center for Nurses, Incorporated Foundation of Tokiwa-Kai, Iwaki, Japan; 10 St. Olive Nursing Home, Shirakawa, Japan; 11 Department of Nursing, Fukushima Medical University Hospital, Fukushima, Japan; 12 Department of Psychiatry, Hori Mental Clinic, Minamisoma, Japan; 13 Department of Breast and Thyroid Surgery, Jyoban Hospital of Tokiwa Foundation, Iwaki, Japan; 14 Department of Neurosurgery, Minamisoma Municipal General Hospital, Minamisoma, Japan; 15 Department of Psychiatry, Aizu Medical Center, Fukushima Medical University, Aizuwakamatsu, Japan

**Keywords:** Public health, Statistics

## Abstract

The medical situation during disasters often differs from that at usual times. Disasters can lead to significant mortality that can be difficult to monitor. The types of disaster-related deaths are largely unknown. In this study, we conducted a survey to categorize the disaster-related deaths caused by a radiation disaster. A total of 520 people living in Minamisoma City, Fukushima Prefecture, at the time of the Fukushima Daiichi Nuclear Power Plant accident, who were certified to have died due to disaster-related causes were surveyed. We divided the participants into those who were at home at the time of the earthquake and those who were in hospitals or facilities when the disaster struck and conducted a hierarchical cluster analysis of the two groups. Disaster-related deaths could be divided into seven groups for those who were at home at the time of the disaster and five groups for those who were in hospitals or facilities at the time of the disaster. Each group showed different characteristics, such as "the group with disabilities," "the group receiving care," and "the group with depression," and it became evident that not only uniform post-disaster support, but support tailored to the characteristics of each group is necessary.

## Introduction

People experience long-term consequences in the event of a disaster^1–3^. Since disasters can have a tremendous impact on healthcare, it is important to identify the challenges experienced by the healthcare sector in times of disaster and take appropriate steps to address them^4–6^.

Disaster-related deaths are among the most important issues in disaster medicine and can be caused by secondary health effects of a disaster, such as emergency evacuation, relocation, shelter environment, disruption of healthcare delivery services, and psychosocial effects^7^. The environment of a disaster-stricken area during and after a disaster differs from that of normal times in several ways, and thus interventions for the resulting problems require a different approach^8^. Previous studies have reported that disaster-related deaths are caused by stress, fatigue, and worsening of pre-existing medical conditions due to long-term evacuation. Moreover, it has also been reported that these disaster-related deaths are more likely to occur in older adults in their 70 s to 80 s, with no significant sex bias, and among those requiring nursing care. Respiratory diseases have been proclaimed to be the most common direct cause of disaster-related deaths^9,10^. However, the concept of disaster-related deaths itself is still new, and information is limited, necessitating further clarification.

Following the March 2011 Great East Japan Earthquake and the subsequent tsunami and accident at the Fukushima Daiichi Nuclear Power Plant (FDNPP), evacuation orders were issued for large areas of the coastal region of the Fukushima Prefecture (Soma and Futaba areas), where the FDNPP is located. Hospitals around the nuclear power plant lost inpatients because they were unable to provide adequate care due to the reduction in medical staff and disconnection of medical infrastructure during the evacuation^11–22^. Indirect health effects, such as weakened early response medical systems, reduced access to medical care, changes in living conditions, and psychological and physical stress after an accident, have been reported to increase the mortality rate^23–31^. Although capturing the nature of these disaster-related deaths is important for preventing casualties in large-scale disasters, no detailed analysis has been conducted to identify the characteristics of disaster-related deaths among populations that died due to radiation disasters.

However, in non-standard cohort studies, such as disaster or pandemic studies, the methods of participant enrollment and data collection may differ from those of regular cohorts, thereby making it difficult to directly determine the overall characteristics from the collected data^32–34^. Therefore, to understand the overall characteristics of disaster-related deaths, it is important to develop a typology of these types of death cases and categorize each death into a group of similar type of cases.

This study aimed to clarify the characteristic patterns of disaster-related deaths in a radiation disaster by grouping similar case groups among those who died in Minamisoma City, where some areas were included in the evacuation-ordered zone after the FDNPP accident. Identifying the typical patterns of disaster-related deaths in a major type of disaster will help gain more in-depth understanding of the nature of disaster-related deaths.

## Materials and methods

### Study design and setting

This retrospective observational study was conducted in Minamisoma City, Fukushima Prefecture, and included cases that were certified as disaster-related deaths between September 2011 and February 2021.

**Figure 1:**
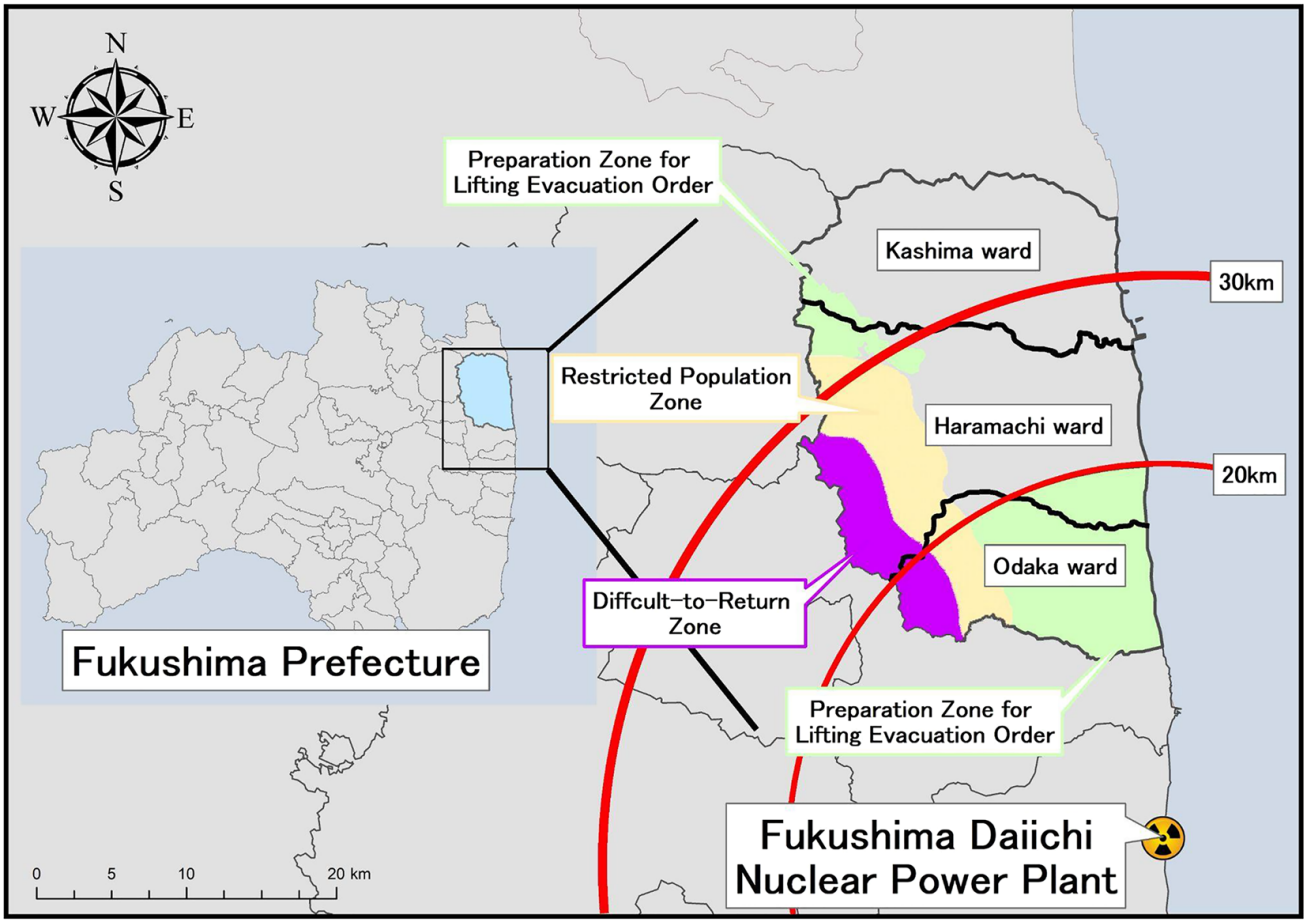
Location of Minamisoma City and Fukushima Daiichi Nuclear Power Plant along with the “Preparation Zone for Lifting Evacuation Order,” “Restricted Population Zone,” and “Difficult-to-Return Zone” in Minamisoma City from April 1, 2012, to July 12, 2016. This map was created with ESRI's Arc GIS pro version 3.1. (https://www.esrij.com/products/arcgis).

Minamisoma City is located 11–38 km north of FDNPP. Immediately after the FDNPP accident that occurred following the earthquake, the Japanese government issued an evacuation order within a 20 km radius and an indoor evacuation order within a 20–30 km radius. A few weeks after the accident, the Japanese government divided Minamisoma City into four areas. The area within a 20-km radius of the FDNPP was designated as the evacuation zone, the western part of the city outside the 20-km radius as the planned evacuation zone, the 20–30 km radius of the FDNPP as the emergency evacuation preparation zone, and the area beyond the 30-km radius of the FDNPP as the non-evacuation zone. Odaka Ward in the southern part of Minamisoma City was designated as the evacuation zone because it is located within 20 km of the FDNPP. The evacuation order was temporarily changed to "Preparation Zone for Lifting Evacuation Order," "Restricted Population Zone," and "Difficult-to-Return Zone." Finally, on July 12, 2016, the evacuation order was lifted with the exception of "Difficult-to-Return Zone”^35^ (Fig. 1).

The study included a total of 520 people who lived in Minamisoma City on March 11, 2011, the day the Great East Japan Earthquake occurred, and whose deaths were certified as disaster-related by the Minamisoma City Committee for Certification of Disaster-Related Deaths. Disaster-related deaths in Japan are certified by the committee after an examination performed by the municipality’s certification committee based on an application form submitted by the bereaved family of the deceased. Therefore, the definition of disaster-related deaths in this study conformed to the criteria used by the Minamisoma City Committee for recognition of disaster-related deaths.

The Minamisoma City Office in Fukushima Prefecture provided us with the application forms and accompanying materials of the 520 targeted people who were certified as having died from disaster-related causes. These application forms and accompanying materials written by the bereaved families of the deceased were used to collect data and create the "Minamisoma City Disaster-Related Death Database" (MCDRD data). The following 24 items without descriptive elements were selected and extracted from the MCDRD data: sex, age at the time of death, evacuation, number of evacuations, number of movements, situation when moving, moving location, accommodation, evacuation housing, household, receiving care certification, disability, increased alcohol consumption, insomnia, depression, dementia, decreased social participation, decreased communication, days since the earthquake, place of death, March 11 residence classification (< 20 km, < 30 km), March 11 hospitalization or inpatient status, March 11 ward of residence, and direct cause of death. The MCDRD data used in this study are not publicly available as they contain personal information and were provided for sole use in our series of studies.

### Data analysis

#### Organize background information

In this study, the causes of death were classified according to the International Classification of Diseases 10th Revision (ICD-10) to make the causes of death statistically tractable. Direct causes of death were substituted for those without a description of the cause of death. For individuals with more than one listed cause of death, multiple authors consulted each other, considered other descriptions of the circumstances at the time of death, and selected the most appropriate cause.

After replacing the cause of death with the ICD-10 code, basic statistics for each variable were calculated. Means and variances were calculated for continuous variables and the number in each category was counted for categorical data (Table 1).

**Table 1:** Summary of background data of the study population.

Variable	Category	Count/Mean
		N = 520
Sex	Female	253 (48.7)
	Male	267 (51.3)
Evacuation	Yes	493 (94.8)
	No	27 (5.2)
Situation when moving	Accompanied	293 (56.3)
	Unaccompanied	190 (36.5)
	Miss	37 (7.1)
Moving location	Within the prefecture	157 (30.2)
	Outside the prefecture	334 (64.2)
	Miss	29 (5.6)
Roommate	Yes	321 (61.7)
	No	193 (37.1)
	Miss	6 (1.2)
Evacuation housing	Temporary housing	6 (1.2)
	Temporary facility	2 (0.4)
	Rental housing	57 (11.0)
	Relative's house	84 (16.2)
	Hospital	142 (27.3)
	Home	25 (4.8)
	Facility	167 (32.1)
	Miss	37 (7.1)
Household	Single	55 (10.6)
	Facility	96 (18.5)
	One	136 (26.2)
	Two	149 (28.7)
	Three	74 (14.2)
	Four	6 (1.2)
	Miss	4 (0.8)
Receiving care certification	Yes	272 (52.3)
	No	201 (38.7)
	Miss	47 (9.0)
Disabilities	Yes	56 (10.8)
	No	419 (80.6)
	Miss	45 (8.7)
Increased alcohol consumption	Yes	5 (1.0)
	No	408 (78.5)
	Miss	107 (20.6)
Insomnia	Yes	105 (20.2)
	No	97 (18.7)
	Miss	318 (61.2)
Depression	Yes	243 (46.7)
	No	81 (15.6)
	Miss	196 (37.7)
Dementia	Yes	100 (19.2)
	No	167 (32.1)
	Miss	253 (48.7)
Decreased social participation	Yes	298 (57.3)
	No	104 (20.0)
	Miss	118 (22.7)
Decreased communication	Yes	207 (39.8)
	No	137 (26.3)
	Miss	176 (33.8)
Place of death	Refuge	7 (1.3)
	Hospital	435 (83.7)
	Home	30 (5.8)
	Facility	36 (6.9)
	Miss	11 (2.1)
Residence classification (3/11)	Under20km	400 (76.9)
	Over30km	119 (22.9)
	Miss	1 (0.2)
Hospitalization/inpatient Status (3/11)	Facility	119 (22.9)
	Hospital	148 (28.5)
	Home	252 (48.5)
Ward of residence (3/11)	Odaka	133 (25.6)
	Kashima	56 (10.8)
	Haramachi	321 (61.7)
	Miss	9 (1.7)
Direct cause of death	A00-B99 (infectious disease)	8 (1.5)
	C00-D48 (malignant growth)	78 (15.0)
	D50-D89 (Hematological or Immunological Diseases)	4 (0.8)
	E00-E90 (Endocrine or Metabolic Disease)	1 (0.2)
	G00-G99 (neurological disease)	3 (0.6)
	I00-I99 (circulatory disease)	123 (23.7)
	J00-J99 (respiratory disease)	143 (27.5)
	K00-K93 (gastrointestinal disease)	15 (2.9)
	M00-M99 (musculoskeletal disease)	2 (0.4)
	N00-N99 (urological disease)	25 (4.8)
	R00-R99 (senility)	88 (16.9)
	S00-T98 (Damage or poisoning)	3 (0.6)
	V01-Y98 (Suicide or death by external causes)	19 (3.7)
	Miss	8 (1.5)
Age at the time of death		82.7 ± 11.9
Number of evacuations		2.03 ± 1.50
Number of movements		3.03 ± 2.29
Number of days since the earthquake		230.6 ± 310.2

#### Clustering background information

**Figure 2:**
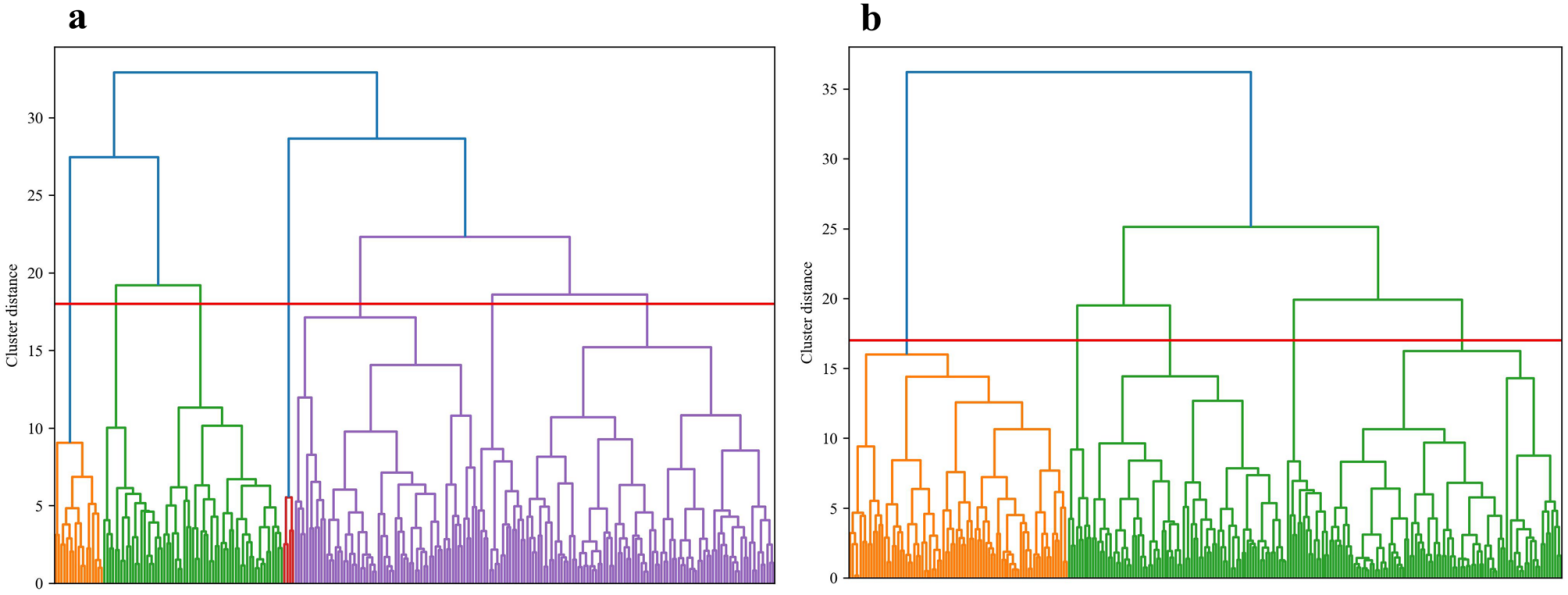
The tree diagrams of hierarchical clustering used to determine the number of clusters. The clustering distance is the distance produced using the Ward method with square Euclidean distance. (**a**) The tree diagram of hierarchical clustering of those who were at home at the time of the earthquake. (**b**) The tree diagram of hierarchical clustering of those who were in the hospital or facility at the time of the earthquake.

**Figure 3:**
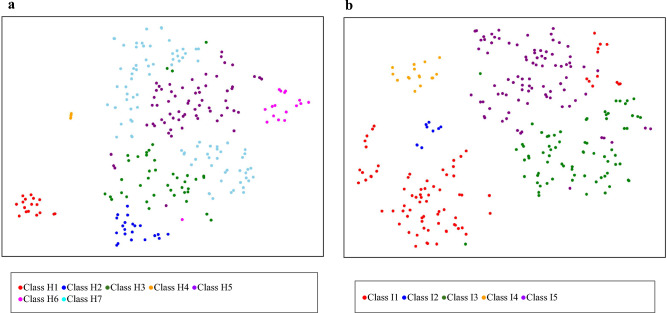
Results of dimensional compression using the t-SNE method and visualization of cluster results in a plan view. (**a**) Visualization of cluster results of those who were at home at the time of the earthquake. (**b**) Visualization of cluster results of those who were in the hospital or facility at the time of the earthquake.

To determine how disaster-related deaths could be classified according to the 24 types of background information, excluding the direct causes of death, a hierarchical cluster analysis was performed on the background information using the Ward method^36^ with square Euclidean distance. Previous studies in Fukushima have revealed differences in the characteristics of those who died at home compared to those who died in hospitals or institutions^14,15,37^. Therefore, statistical analysis was performed to compare the background information between those who died at home and those who died in a hospital or institution, and significant differences were found (Supplemental Table 1). Hierarchical cluster analysis was conducted separately for those who were at home and those who were in hospitals or institutions at the time of the earthquake. One person whose residence at the time of the earthquake was unknown was excluded from the analysis. While conducting the analysis, there were 17 items of binary data and continuous variables: sex, age at the time of death, whether or not the person was evacuated, number of times evacuated, number of times moved, situation at the time of move, location of move, family situation (which meant single at the time of death or not), disability, whether or not the person was receiving nursing care at the time of the disaster, increased drinking opportunities and alcohol consumption, insomnia, depressed mood, development or severity of dementia, decreased social participation and activities, decreased communication, and date since disaster. Missing values were added using the multiple assignment method. Each variable was standardized (Z-score) to ensure that the data scale was uniform. The number of clusters was determined by combining the tree diagrams (Fig. 2), which were created when the Ward method was implemented, and the interpretability of the results was good. After clustering, color coding was performed for each cluster class on the dimensionality-reduced data using the t-SNE^38^ algorithm to visualize the results (Fig. 3).

#### Analysis of cluster results and their factors

**Figure 4:**
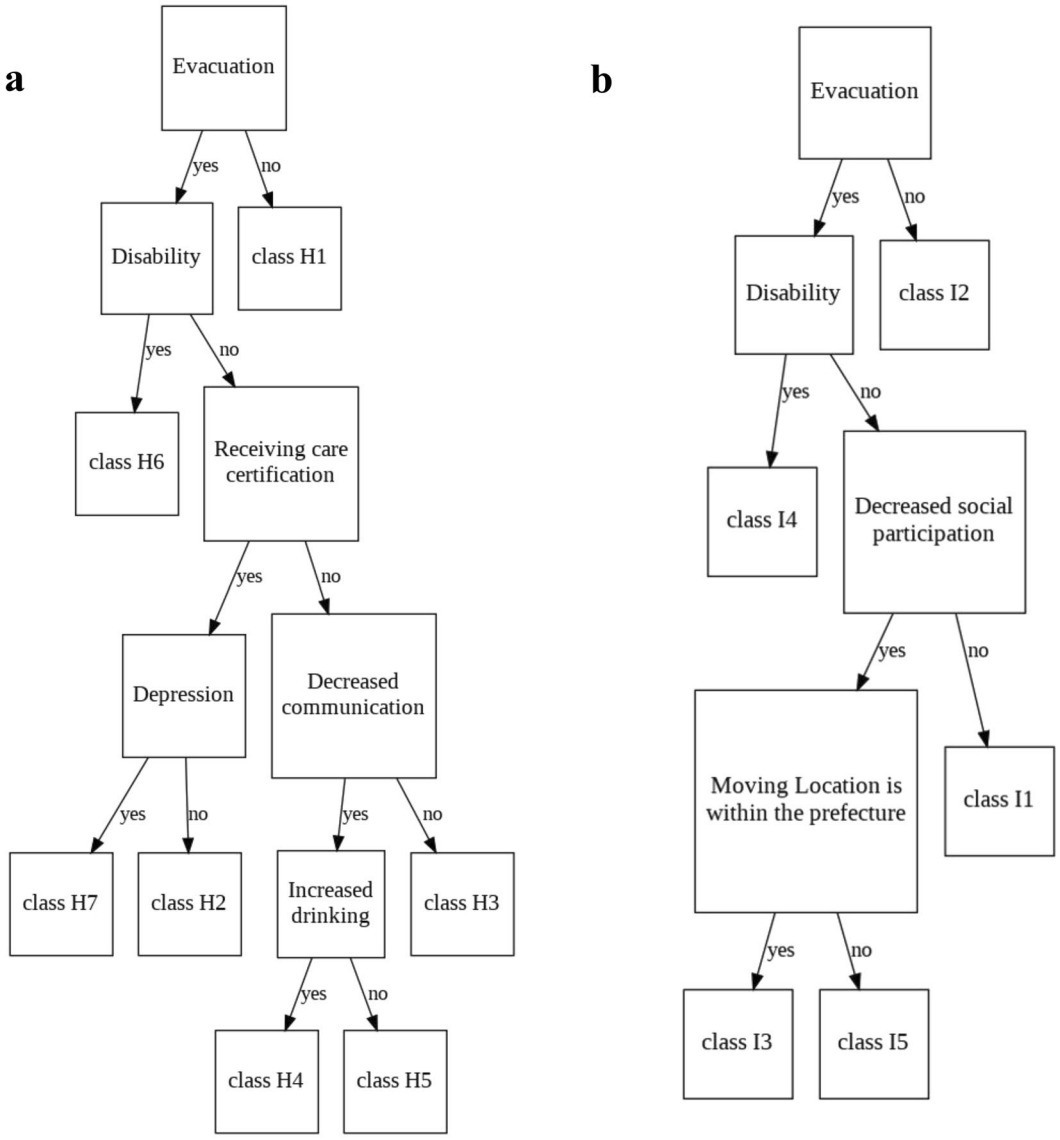
Main factors revealed by cluster analysis and the classification algorithm graphs that used them. (**a**) Classification algorithm graph for those who were at home at the time of the earthquake. (**b**) Classification algorithm graph for those who were in the hospital or facility at the time of the earthquake.

To clarify the relationship between the background information and the degree to which the results of the cluster performed earlier were influenced by the factors, a table summarizing the relationship between each class and variable was created (Tables 2 and 3). Categorical variables were counted by category, and continuous variables were derived as means and variances. The number of participants before cluster analysis was sufficient, but some clusters had few participants, and these were considered the minority. In addition, to clarify the variables that contributed to the cluster results, the Fisher’s exact method was applied to the cluster results for categorical variables, one-way analysis of variance (ANOVA) was applied to continuous variables, and effect sizes were derived. V ≥ 0.50 and η^2^ ≥ 0.14 were determined to have a large effect size^39^. Fisher’s exact test or one-way ANOVA was performed only for groups with 30 or more participants. In contrast, descriptive statistics were used for minority groups with less than 30 participants. Finally, the experts discussed the results and created a graph to interpret the relationship between the cluster results and background factors. (Fig. 4).

**Table 2:** Results of cluster analysis of study participants who were at home at the time of the earthquake.

Variable	Category	Class H1	Class H2	Class H3	Class H4	Class H5	Class H6	Class H7	Total	P value*	Effect sizes^†^
N		17 (6.7)	21 (8.3)	42 (16.7)	4 (1.6)	64 (25.4)	17 (6.7)	87 (34.5)	252		
Sex	Female	6 (35.3)	13 (61.9)	4 (9.5)	0 (0.0)	21 (32.8)	7 (41.2)	50 (57.5)	101 (40.1)	< 0.001	0.39
	Male	11 (64.7)	8 (38.1)	38 (90.5)	4 (100.0)	43 (67.2)	10 (58.8)	37 (42.5)	151 (59.9)		
Evacuation	Yes	0 (0.0)	21 (100.0)	42 (100.0)	4 (100.0)	64 (100.0)	17 (100.0)	87 (100.0)	235 (93.3)	NaN	NaN
	No	17 (100.0)	0 (0.0)	0 (0.0)	0 (0.0)	0 (0.0)	0 (0.0)	0 (0.0)	17 (6.7)		
Situation when moving	Accompanied	2 (11.8)	16 (76.2)	40 (95.2)	3 (75.0)	63 (98.4)	16 (94.1)	78 (89.7)	218 (86.5)	0.11	0.15
	Unaccompanied	0 (0.0)	2 (9.5)	2 (4.8)	1 (25.0)	1 (1.6)	0 (0.0)	8 (9.2)	14 (5.6)		
	Miss	15 (88.2)	3 (14.3)	0 (0.0)	0 (0.0)	0 (0.0)	1 (5.9)	1 (1.1)	20 (7.9)		
Moving location	Within the prefecture	1 (5.9)	7 (33.3)	19 (45.2)	0 (0.0)	7 (10.9)	7 (41.2)	42 (48.3)	83 (32.9)	< 0.001	0.36
	Outside the prefecture	0 (0.0)	12 (57.1)	23 (54.8)	4 (100.0)	57 (89.1)	10 (58.8)	45 (51.7)	151 (59.9)		
	Miss	16 (94.1)	2 (9.5)	0 (0.0)	0 (0.0)	0 (0.0)	0 (0.0)	0 (0.0)	18 (7.1)		
Roommate	Yes	14 (82.4)	19 (90.5)	40 (95.2)	3 (75.0)	56 (87.5)	13 (76.5)	63 (72.4)	208 (82.5)	0.002	0.26
	No	2 (11.8)	2 (9.5)	2 (4.8)	1 (25.0)	7 (10.9)	2 (11.8)	24 (27.6)	40 (15.9)		
	Miss	1 (5.9)	0 (0.0)	0 (0.0)	0 (0.0)	1 (1.6)	2 (11.8)	0 (0.0)	4 (1.6)		
Evacuation housing	Temporary housing	0 (0.0)	0 (0.0)	3 (7.1)	1 (25.0)	0 (0.0)	1 (5.9)	1 (1.1)	6 (2.4)	0.02	0.27
	Temporary facility	0 (0.0)	0 (0.0)	2 (4.8)	0 (0.0)	0 (0.0)	0 (0.0)	0 (0.0)	2 (0.8)		
	Rental housing	0 (0.0)	3 (14.3)	7 (16.7)	1 (25.0)	22 (34.4)	4 (23.5)	15 (17.2)	52 (20.6)		
	Relative's house	0 (0.0)	4 (19.0)	14 (33.3)	0 (0.0)	18 (28.1)	3 (17.6)	28 (32.2)	67 (26.6)		
	Hospital	0 (0.0)	6 (28.6)	4 (9.5)	0 (0.0)	4 (6.2)	0 (0.0)	8 (9.2)	22 (8.7)		
	Home	6 (35.3)	2 (9.5)	4 (9.5)	0 (0.0)	1 (1.6)	0 (0.0)	6 (6.9)	19 (7.5)		
	Facility	0 (0.0)	2 (9.5)	6 (14.3)	1 (25.0)	16 (25.0)	8 (47.1)	24 (27.6)	57 (22.6)		
	Miss	11 (64.7)	4 (19.0)	2 (4.8)	1 (25.0)	3 (4.7)	1 (5.9)	5 (5.7)	27 (10.7)		
Household	Single	3 (17.6)	1 (4.8)	3 (7.1)	0 (0.0)	4 (6.2)	1 (5.9)	7 (8.0)	19 (7.5)	0.48	0.16
	Facility	0 (0.0)	1 (4.8)	1 (2.4)	0 (0.0)	3 (4.7)	0 (0.0)	8 (9.2)	13 (5.2)		
	One	8 (47.1)	7 (33.3)	21 (50.0)	2 (50.0)	23 (35.9)	4 (23.5)	27 (31.0)	92 (36.5)		
	Two	3 (17.6)	7 (33.3)	14 (33.3)	0 (0.0)	21 (32.8)	6 (35.3)	31 (35.6)	82 (32.5)		
	Three	3 (17.6)	3 (14.3)	3 (7.1)	2 (50.0)	12 (18.8)	4 (23.5)	14 (16.1)	41 (16.3)		
	Four	0 (0.0)	0 (0.0)	0 (0.0)	0 (0.0)	1 (1.6)	1 (5.9)	0 (0.0)	2 (0.8)		
	Miss	0 (0.0)	2 (9.5)	0 (0.0)	0 (0.0)	0 (0.0)	1 (5.9)	0 (0.0)	3 (1.2)		
Receiving care certification	Yes	3 (17.6)	15 (71.4)	12 (28.6)	0 (0.0)	7 (10.9)	8 (47.1)	45 (51.7)	90 (35.7)	< 0.001	0.44
	No	12 (70.6)	5 (23.8)	26 (61.9)	4 (100.0)	56 (87.5)	8 (47.1)	32 (36.8)	143 (56.7)		
	Miss	2 (11.8)	1 (4.8)	4 (9.5)	0 (0.0)	1 (1.6)	1 (5.9)	10 (11.5)	19 (7.5)		
Disabilities	Yes	1 (5.9)	5 (23.8)	0 (0.0)	0 (0.0)	3 (4.7)	17 (100.0)	0 (0.0)	26 (10.3)	0.06	0.17
	No	16 (94.1)	15 (71.4)	40 (95.2)	4 (100.0)	61 (95.3)	0 (0.0)	74 (85.1)	210 (83.3)		
	Miss	0 (0.0)	1 (4.8)	2 (4.8)	0 (0.0)	0 (0.0)	0 (0.0)	13 (14.9)	16 (6.3)		
Increased alcohol consumption	Yes	0 (0.0)	0 (0.0)	0 (0.0)	4 (100.0)	0 (0.0)	0 (0.0)	0 (0.0)	4 (1.6)	NaN	NaN
	No	11 (64.7)	18 (85.7)	28 (66.7)	0 (0.0)	37 (57.8)	10 (58.8)	64 (73.6)	168 (66.7)		
	Miss	6 (35.3)	3 (14.3)	14 (33.3)	0 (0.0)	27 (42.2)	7 (41.2)	23 (26.4)	80 (31.7)		
Insomnia	Yes	3 (17.6)	1 (4.8)	10 (23.8)	3 (75.0)	29 (45.3)	7 (41.2)	26 (29.9)	79 (31.3)	< 0.001	0.48
	No	2 (11.8)	17 (81.0)	7 (16.7)	0 (0.0)	1 (1.6)	0 (0.0)	1 (1.1)	28 (11.1)		
	Miss	12 (70.6)	3 (14.3)	25 (59.5)	1 (25.0)	34 (53.1)	10 (58.8)	60 (69.0)	145 (57.5)		
Depression	Yes	10 (58.8)	0 (0.0)	23 (54.8)	3 (75.0)	49 (76.6)	15 (88.2)	55 (63.2)	155 (61.5)	NaN	NaN
	No	2 (11.8)	16 (76.2)	0 (0.0)	0 (0.0)	0 (0.0)	0 (0.0)	0 (0.0)	18 (7.1)		
	Miss	5 (29.4)	5 (23.8)	19 (45.2)	1 (25.0)	15 (23.4)	2 (11.8)	32 (36.8)	79 (31.3)		
Dementia	Yes	0 (0.0)	0 (0.0)	0 (0.0)	0 (0.0)	22 (34.4)	6 (35.3)	23 (26.4)	51 (20.2)	< 0.001	0.61
	No	8 (47.1)	18 (85.7)	33 (78.6)	1 (25.0)	12 (18.8)	4 (23.5)	12 (13.8)	88 (34.9)		
	Miss	9 (52.9)	3 (14.3)	9 (21.4)	3 (75.0)	30 (46.9)	7 (41.2)	52 (59.8)	113 (44.8)		
Decreased social participation	Yes	12 (70.6)	2 (9.5)	22 (52.4)	4 (100.0)	54 (84.4)	15 (88.2)	68 (78.2)	177 (70.2)	< 0.001	0.38
	No	4 (23.5)	16 (76.2)	10 (23.8)	0 (0.0)	4 (6.2)	0 (0.0)	1 (1.1)	35 (13.9)		
	Miss	1 (5.9)	3 (14.3)	10 (23.8)	0 (0.0)	6 (9.4)	2 (11.8)	18 (20.7)	40 (15.9)		
Decreased communication	Yes	5 (29.4)	0 (0.0)	1 (2.4)	3 (75.0)	41 (64.1)	12 (70.6)	47 (54.0)	109 (43.3)	< 0.001	0.82
	No	7 (41.2)	19 (90.5)	31 (73.8)	0 (0.0)	4 (6.2)	2 (11.8)	5 (5.7)	68 (27.0)		
	Miss	5 (29.4)	2 (9.5)	10 (23.8)	1 (25.0)	19 (29.7)	3 (17.6)	35 (40.2)	75 (29.8)		
Place of death	Hospital	9 (52.9)	17 (81.0)	35 (83.3)	3 (75.0)	53 (82.8)	13 (76.5)	77 (88.5)	207 (82.1)	0.44	0.12
	Home	8 (47.1)	2 (9.5)	5 (11.9)	1 (25.0)	5 (7.8)	2 (11.8)	3 (3.4)	26 (10.3)		
	Refuge	0 (0.0)	2 (9.5)	0 (0.0)	0 (0.0)	2 (3.1)	0 (0.0)	3 (3.4)	7 (2.8)		
	Facility	0 (0.0)	0 (0.0)	2 (4.8)	0 (0.0)	1 (1.6)	0 (0.0)	3 (3.4)	6 (2.4)		
	Miss	0 (0.0)	0 (0.0)	0 (0.0)	0 (0.0)	3 (4.7)	2 (11.8)	1 (1.1)	6 (2.4)		
Residence classification (3/11)	Over30km	5 (29.4)	3 (14.3)	5 (11.9)	1 (25.0)	8 (12.5)	3 (17.6)	17 (19.5)	42 (16.7)	0.38	0.10
	Under20km	12 (70.6)	18 (85.7)	37 (88.1)	3 (75.0)	56 (87.5)	14 (82.4)	70 (80.5)	210 (83.3)		
Hospitalization/Inpatient Status (3/11)	Home	17 (100.0)	21 (100.0)	42 (100.0)	4 (100.0)	64 (100.0)	17 (100.0)	87 (100.0)	252 (100.0)	NaN	NaN
Ward of residence (3/11)	Haramachi	12 (70.6)	16 (76.2)	27 (64.3)	4 (100.0)	29 (45.3)	13 (76.5)	45 (51.7)	146 (57.9)	0.11	0.14
	Odaka	2 (11.8)	4 (19.0)	12 (28.6)	0 (0.0)	32 (50.0)	2 (11.8)	31 (35.6)	83 (32.9)		
	Kashima	3 (17.6)	1 (4.8)	3 (7.1)	0 (0.0)	3 (4.7)	2 (11.8)	11 (12.6)	23 (9.1)		
Direct cause of death	A00-B99 (infectious disease)	1 (5.9)	0 (0.0)	1 (2.4)	0 (0.0)	0 (0.0)	2 (11.8)	1 (1.1)	5 (2.0)	0.08	NaN
	C00-D48 (malignant growth)	1 (5.9)	1 (4.8)	15 (35.7)	1 (25.0)	21 (32.8)	3 (17.6)	17 (19.5)	59 (23.4)		
	D50-D89 (Hematological or Immunological Diseases)	0 (0.0)	0 (0.0)	0 (0.0)	0 (0.0)	2 (3.1)	0 (0.0)	0 (0.0)	2 (0.8)		
	E00-E90 (Endocrine or Metabolic Disease)	0 (0.0)	0 (0.0)	0 (0.0)	0 (0.0)	0 (0.0)	1 (5.9)	0 (0.0)	1 (0.4)		
	I00-I99 (circulatory disease)	5 (29.4)	12 (57.1)	5 (11.9)	0 (0.0)	10 (15.6)	4 (23.5)	26 (29.9)	62 (24.6)		
	J00-J99 (respiratory disease)	3 (17.6)	3 (14.3)	10 (23.8)	0 (0.0)	14 (21.9)	1 (5.9)	20 (23.0)	51 (20.2)		
	K00-K93 (gastrointestinal disease)	0 (0.0)	0 (0.0)	3 (7.1)	1 (25.0)	1 (1.6)	0 (0.0)	2 (2.3)	7 (2.8)		
	N00-N99 (urological disease)	0 (0.0)	1 (4.8)	2 (4.8)	1 (25.0)	1 (1.6)	1 (5.9)	3 (3.4)	9 (3.6)		
	R00-R99 (senility)	6 (35.3)	4 (19.0)	4 (9.5)	0 (0.0)	6 (9.4)	3 (17.6)	14 (16.1)	37 (14.7)		
	S00-T98 (Damage or poisoning)	0 (0.0)	0 (0.0)	0 (0.0)	0 (0.0)	1 (1.6)	1 (5.9)	0 (0.0)	2 (0.8)		
	V01-Y98 (Suicide or death by external causes)	1 (5.9)	0 (0.0)	2 (4.8)	1 (25.0)	8 (12.5)	1 (5.9)	4 (4.6)	17 (6.7)		
Age at the time of death		81.2 ± 10.3	85.9 ± 9.24	78.6 ± 10.4	61.8 ± 22.9	75.3 ± 18.2	77.2 ± 10.8	85.8 ± 9.44	80.7 ± 13.6	< 0.001	0.12
Number of evacuations		0.0588 ± 0.243	1.43 ± 0.87	2.44 ± 1.47	2.75 ± 0.957	3.80 ± 1.92	2.24 ± 1.09	2.17 ± 1.14	2.44 ± 1.69	< 0.001	0.19
Number of movements		0.118 ± 0.332	2.10 ± 1.26	4.10 ± 2.52	3.50 ± 1.73	5.53 ± 2.79	3.18 ± 1.70	3.44 ± 1.68	3.73 ± 2.51	< 0.001	0.14
Number of days since the earthquake		98.0 ± 172.3	51.7 ± 39.2	228.8 ± 225.0	667.5 ± 237.6	611.5 ± 514.2	488.1 ± 432.9	192.0 ± 137.6	314.2 ± 371.0	< 0.001	0.26

*Fisher’s test for categorical variables, one-way ANOVA otherwise.

^†^Cramer's V for categorical variables, η^2^ otherwise.

Statistical analysis is performed only for groups with n ≥ 30.

**Table 3:** Results of cluster analysis of those who were in hospital or facility at the time of the earthquake.

Variables	Category	Class I1	Class I2	Class I3	Class I4	Class I5	Total	p value*	Effect sizes^†^
N		82 (30.1)	9 (3.4)	73 (27.3)	16 (6.0)	87 (32.6)	267		
Sex	Female	53 (64.6)	5 (55.6)	40 (54.8)	5 (31.2)	48 (55.2)	151 (56.6)	0.36	0.09
	Male	29 (35.4)	4 (44.4)	33 (45.2)	11 (68.8)	39 (44.8)	116 (43.4)		
Evacuation	Yes	82 (100.0)	0 (0.0)	73 (100.0)	16 (100.0)	87 (100.0)	258 (96.6)	NaN	NaN
	No	0 (0.0)	9 (100.0)	0 (0.0)	0 (0.0)	0 (0.0)	9 (3.4)		
Situation when moving	Accompanied	21 (25.6)	3 (33.3)	36 (49.3)	3 (18.8)	12 (13.8)	75 (28.1)	< 0.001	0.35
	Unaccompanied	57 (69.5)	1 (11.1)	31 (42.5)	13 (81.2)	73 (83.9)	175 (65.5)		
	Miss	4 (4.9)	5 (55.6)	6 (8.2)	0 (0.0)	2 (2.3)	17 (6.4)		
Moving location	Within the prefecture	20 (24.4)	4 (44.4)	42 (57.5)	2 (12.5)	6 (6.9)	74 (27.7)	< 0.001	0.48
	Outside the prefecture	59 (72.0)	0 (0.0)	28 (38.4)	14 (87.5)	81 (93.1)	182 (68.2)		
	Miss	3 (3.7)	5 (55.6)	3 (4.1)	0 (0.0)	0 (0.0)	11 (4.1)		
Roommate	Yes	28 (34.1)	5 (55.6)	53 (72.6)	9 (56.2)	17 (19.5)	112 (41.9)	< 0.001	0.46
	No	54 (65.9)	4 (44.4)	18 (24.7)	7 (43.8)	70 (80.5)	153 (57.3)		
	Miss	0 (0.0)	0 (0.0)	2 (2.7)	0 (0.0)	0 (0.0)	2 (0.7)		
Evacuation housing	Rental housing	1 (1.2)	0 (0.0)	2 (2.7)	0 (0.0)	2 (2.3)	5 (1.9)	0.13	0.15
	Relative's house	3 (3.7)	0 (0.0)	7 (9.6)	1 (6.2)	6 (6.9)	17 (6.4)		
	Hospital	37 (45.1)	3 (33.3)	38 (52.1)	7 (43.8)	34 (39.1)	119 (44.6)		
	Home	1 (1.2)	3 (33.3)	2 (2.7)	0 (0.0)	0 (0.0)	6 (2.2)		
	Facility	37 (45.1)	0 (0.0)	21 (28.8)	8 (50.0)	44 (50.6)	110 (41.2)		
	Miss	3 (3.7)	3 (33.3)	3 (4.1)	0 (0.0)	1 (1.1)	10 (3.7)		
Household	Single	15 (18.3)	1 (11.1)	4 (5.5)	0 (0.0)	16 (18.4)	36 (13.5)	< 0.001	0.26
	Facility	33 (40.2)	2 (22.2)	10 (13.7)	6 (37.5)	32 (36.8)	83 (31.1)		
	One	11 (13.4)	2 (22.2)	15 (20.5)	3 (18.8)	12 (13.8)	43 (16.1)		
	Two	15 (18.3)	2 (22.2)	26 (35.6)	4 (25.0)	20 (23.0)	67 (25.1)		
	Three	7 (8.5)	2 (22.2)	16 (21.9)	3 (18.8)	5 (5.7)	33 (12.4)		
	Four	1 (1.2)	0 (0.0)	2 (2.7)	0 (0.0)	1 (1.1)	4 (1.5)		
	Miss	0 (0.0)	0 (0.0)	0 (0.0)	0 (0.0)	1 (1.1)	1 (0.4)		
Receiving care certification	Yes	61 (74.4)	4 (44.4)	37 (50.7)	12 (75.0)	67 (77.0)	181 (67.8)	0.002	0.25
	No	13 (15.9)	5 (55.6)	24 (32.9)	4 (25.0)	12 (13.8)	58 (21.7)		
	Miss	8 (9.8)	0 (0.0)	12 (16.4)	0 (0.0)	8 (9.2)	28 (10.5)		
Disabilities	Yes	13 (15.9)	0 (0.0)	0 (0.0)	16 (100.0)	0 (0.0)	29 (10.9)	< 0.001	0.34
	No	63 (76.8)	9 (100.0)	59 (80.8)	0 (0.0)	78 (89.7)	209 (78.3)		
	Miss	6 (7.3)	0 (0.0)	14 (19.2)	0 (0.0)	9 (10.3)	29 (10.9)		
Increased alcohol consumption	Yes	0 (0.0)	0 (0.0)	0 (0.0)	0 (0.0)	1 (1.1)	1 (0.4)	0.64	0.09
	No	80 (97.6)	7 (77.8)	63 (86.3)	14 (87.5)	75 (86.2)	239 (89.5)		
	Miss	2 (2.4)	2 (22.2)	10 (13.7)	2 (12.5)	11 (12.6)	27 (10.1)		
Insomnia	Yes	1 (1.2)	1 (11.1)	6 (8.2)	6 (37.5)	12 (13.8)	26 (9.7)	< 0.001	0.77
	No	56 (68.3)	3 (33.3)	7 (9.6)	0 (0.0)	2 (2.3)	68 (25.5)		
	Miss	25 (30.5)	5 (55.6)	60 (82.2)	10 (62.5)	73 (83.9)	173 (64.8)		
Depression	Yes	7 (8.5)	2 (22.2)	21 (28.8)	11 (68.8)	47 (54.0)	88 (33.0)	< 0.001	0.86
	No	56 (68.3)	3 (33.3)	3 (4.1)	0 (0.0)	0 (0.0)	62 (23.2)		
	Miss	19 (23.2)	4 (44.4)	49 (67.1)	5 (31.2)	40 (46.0)	117 (43.8)		
Dementia	Yes	13 (15.9)	0 (0.0)	7 (9.6)	8 (50.0)	21 (24.1)	49 (18.4)	< 0.001	0.49
	No	51 (62.2)	3 (33.3)	16 (21.9)	2 (12.5)	6 (6.9)	78 (29.2)		
	Miss	18 (22.0)	6 (66.7)	50 (68.5)	6 (37.5)	60 (69.0)	140 (52.4)		
Decreased social participation	Yes	18 (22.0)	3 (33.3)	26 (35.6)	13 (81.2)	61 (70.1)	121 (45.3)	< 0.001	0.73
	No	59 (72.0)	3 (33.3)	6 (8.2)	0 (0.0)	0 (0.0)	68 (25.5)		
	Miss	5 (6.1)	3 (33.3)	41 (56.2)	3 (18.8)	26 (29.9)	78 (29.2)		
Decreased communication	Yes	12 (14.6)	2 (22.2)	20 (27.4)	14 (87.5)	50 (57.5)	98 (36.7)	< 0.001	0.71
	No	56 (68.3)	2 (22.2)	6 (8.2)	0 (0.0)	4 (4.6)	68 (25.5)		
	Miss	14 (17.1)	5 (55.6)	47 (64.4)	2 (12.5)	33 (37.9)	101 (37.8)		
Place of death	Facility	12 (14.6)	0 (0.0)	4 (5.5)	3 (18.8)	11 (12.6)	30 (11.2)	0.36	0.09
	Hospital	69 (84.1)	9 (100.0)	63 (86.3)	12 (75.0)	75 (86.2)	228 (85.4)		
	Home	1 (1.2)	0 (0.0)	2 (2.7)	0 (0.0)	1 (1.1)	4 (1.5)		
	Miss	0 (0.0)	0 (0.0)	4 (5.5)	1 (6.2)	0 (0.0)	5 (1.9)		
Residence classification (3/11)	Over30km	35 (42.7)	1 (11.1)	21 (28.8)	6 (37.5)	14 (16.1)	77 (28.8)	< 0.001	0.24
	Under20km	47 (57.3)	8 (88.9)	52 (71.2)	10 (62.5)	73 (83.9)	190 (71.2)		
Hospitalization/Inpatient Status (3/11)	Facility	40 (48.8)	3 (33.3)	17 (23.3)	8 (50.0)	51 (58.6)	119 (44.6)	< 0.001	0.29
	Hospital	42 (51.2)	6 (66.7)	56 (76.7)	8 (50.0)	36 (41.4)	148 (55.4)		
Ward of residence (3/11)	Haramachi	59 (72.0)	8 (88.9)	50 (68.5)	8 (50.0)	50 (57.5)	175 (65.5)	0.04	0.15
	Odaka	13 (15.9)	1 (11.1)	7 (9.6)	5 (31.2)	24 (27.6)	50 (18.7)		
	Kashima	10 (12.2)	0 (0.0)	12 (16.4)	3 (18.8)	8 (9.2)	33 (12.4)		
	Miss	0 (0.0)	0 (0.0)	4 (5.5)	0 (0.0)	5 (5.7)	9 (3.4)		
Direct cause of death	A00-B99 (infectious disease)	1 (1.2)	0 (0.0)	2 (2.7)	0 (0.0)	0 (0.0)	3 (1.1)	0.39	NaN
	C00-D48 (malignant growth)	8 (9.8)	0 (0.0)	5 (6.8)	0 (0.0)	6 (6.9)	19 (7.1)		
	D50-D89 (Hematological or Immunological Diseases)	0 (0.0)	1 (11.1)	1 (1.4)	0 (0.0)	0 (0.0)	2 (0.7)		
	G00-G99 (neurological disease)	1 (1.2)	0 (0.0)	0 (0.0)	0 (0.0)	2 (2.3)	3 (1.1)		
	I00-I99 (circulatory disease)	23 (28.0)	1 (11.1)	10 (13.7)	5 (31.2)	22 (25.3)	61 (22.8)		
	J00-J99 (respiratory disease)	28 (34.1)	2 (22.2)	23 (31.5)	4 (25.0)	35 (40.2)	92 (34.5)		
	K00-K93 (gastrointestinal disease)	1 (1.2)	1 (11.1)	3 (4.1)	0 (0.0)	3 (3.4)	8 (3.0)		
	M00-M99 (musculoskeletal disease)	0 (0.0)	0 (0.0)	1 (1.4)	0 (0.0)	1 (1.1)	2 (0.7)		
	N00-N99 (urological disease)	5 (6.1)	1 (11.1)	6 (8.2)	1 (6.2)	3 (3.4)	16 (6.0)		
	R00-R99 (senility)	12 (14.6)	3 (33.3)	17 (23.3)	4 (25.0)	14 (16.1)	50 (18.7)		
	S00-T98 (Damage or poisoning)	0 (0.0)	0 (0.0)	0 (0.0)	1 (6.2)	0 (0.0)	1 (0.4)		
	V01-Y98 (Suicide or death by external causes)	2 (2.4)	0 (0.0)	0 (0.0)	0 (0.0)	0 (0.0)	2 (0.7)		
	Miss	1 (1.2)	0 (0.0)	5 (6.8)	1 (6.2)	1 (1.1)	8 (3.0)		
Age at the time of death		85.8 ± 9.2	82.9 ± 14.4	82.1 ± 10.1	79.1 ± 13.3	86.7 ± 7.93	84.6 ± 9.75	0.004	0.04
Number of evacuations		1.38 ± 0.855	0.111 ± 0.333	1.45 ± 0.708	1.88 ± 1.20	2.17 ± 1.49	1.64 ± 1.17	< 0.001	0.10
Number of movements		1.99 ± 1.55	0.333 ± 0.500	1.90 ± 1.27	3.25 ± 1.61	3.21 ± 2.15	2.38 ± 1.83	< 0.001	0.11
Number of days since the earthquake		90.2 ± 95.0	40.1 ± 65.1	94.5 ± 92.5	666.7 ± 500.2	174.6 ± 144.4	151.7 ± 211.3	< 0.001	0.11

*Fisher’s test for categorical variables, one-way ANOVA otherwise.

^†^Cramer's V for categorical variables, η^2^ otherwise.

Statistical analysis is performed only for groups with n ≥ 30.

These analyses were performed using python version 3.9.7.

### Ethics declarations

This study was approved by the Minamisoma Municipal General Hospital Ethics Committee (approval number:2-21) and the Fukushima Medical University Ethics Committee (reference number:2020-297). As data were evaluated retrospectively and pseudonymously and were solely obtained for treatment purposes, the requirement for informed consent was waived by the ethics committees of Minamisoma Municipal General Hospital and the Fukushima Medical University. The study was conducted in accordance with the tenets of the Declaration of Helsinki.

## Results

### Data summary

Of the 520 patients enrolled in the MCDRD data, 267 (51.3%) were male and 253 (48.7%) were female. The mean age of the study population was 83 (range 14–105, median 85, standard deviation 11.9) years. At the time of the earthquake, 272 (52.3%) individuals were receiving long-term care, and 56 (10.8%) had disabilities. The residences of 321 (61.7%), 56 (10.8%), and 133 (25.6%) study participants were in Haramachi, Kashima, and Odaka, respectively. After the earthquake, 243 (46.8%) participants showed symptoms of mood swings or depression, and 493 (94.8%) were evacuated. Dementia worsened in 100 (19.2%) patients, and 105 (20.2%) had insomnia (Table 1).

### Results of each class on the basis of cluster analysis (group that was at home at the time of the earthquake)

A total of 252 people were at home at the time of the disaster, and the nature of these disaster-related deaths could be divided into seven categories: Class H1, 17 (6.7%); Class H2, 21 (8.3%); Class H3, 42 (16.7%); Class H4, 4 (1.6%); Class H5, 64 (25.4%); Class H6, 17 (6.7%); and Class H7, 87 (34.5%). The leading causes of death were cardiovascular diseases, 62 (24.6%); malignant neoplasms, 59 (23.4%); and respiratory diseases, 51 (20.2%) (Table 2).

The characteristics of each cluster, listed in order of number of persons, were as follows. Typical example of Class H7 was "elderly with depression" {age (85.8 ± 9.44 years, p < 0.001, η^2^ = 0.12), depression [yes, 55 (63.2%) and no, 0 (0.0%)], receiving care certification [yes, 45 (51.7%) and no, 32 (36.8%), p < 0.001, V = 0.44], and the number of days from the earthquake to death (192.0 ± 137.6, p < 0.001, η^2^ = 0.26)}. In Class H5, the typical cases included “those who had not received care certification or were not disabled, but had reduced social activity and depressive feelings” {reduced social activity [yes, 54 (84.4%) and no, 4 (6.2%), p < 0.001, V = 0.38], depression [yes, 49 (76.6%) and no, 0 (0.0%)], receiving care certification [yes, 7 (10.9%) and no, 56(87.5%), p < 0.001, V = 0.44], disability [yes, 3 (4.7%) and no, 61 (95.3%), p = 0.06, V = 0.17], and number of days from the earthquake to death (611.5 ± 514.2)}. Typical example of Class H3 comprised "those with no care certification or disability, no impaired communication, but with depressive feelings" {impaired communication [yes, 1 (2.4%) and no, 31 (73.8%), p < 0.001, V = 0.82], depression [yes, 23 (54.8%) and no, 0 (0.0%)], receiving care certification [yes, 12 (28.6%) and no, 26 (61.9%), p < 0.001, V = 0.44], disability [with, 0 (0.0%) and without, 40 (95.2%), p = 0.06, V = 0.17], and number of days from the earthquake to death (228.8 ± 225.0, p < 0.001, η^2^ = 0.26)}. A typical example of class H2 was "elderly, without disability or depression, and those who received care certification" {age (85.9 ± 9.24 years), depression [yes, 0 (0.0%) and no, 16 (76.2%)], receiving care certification [yes, 15 (71.4%) and no, 5 (23.8%)], disability [yes, 5 (23.8%) and no, 15(71.4%)], and number of days from the earthquake to death (51.7 ± 39.2)}. For Class H1, a typical example was "those who did not receive care certification or were not disabled and did not evacuate after the earthquake but stayed at home” { evacuation [yes, 0 (0.0%) and no, 17 (100%)], receiving care certification [yes, 3 (17.6%) and no, 12 (70.6%)], disability [yes, 1 (5.9%) and no, 16 (94.1%)], and the number of days from the earthquake to death (98.0 ± 172.3)}.Typical example of Class H6 comprised those who were “depressed and disabled” {depression [yes, 15 (88.2%) and no, 0 (0.0%)], disability [yes, 17 (100.0%) and no, 0 (0.0%)], and number of days from the disaster to death (488.1 ± 432.9)}. A typical example of Class H4 was "those with depression and increased alcohol consumption" {depression [yes, 3 (75%) and no, 0 (0.0%)], increased alcohol consumption [yes, 4 (100%) and no, 0 (0.0%)], and number of days from the disaster to death (667.5 ± 237.6)}.

### Results of cluster analysis for each class (group that was in a hospital or facility at the time of the earthquake)

A total of 267 people were in hospitals or institutions at the time of the disaster, and the nature of these disaster-related deaths can be divided into five categories: Class I1, 82 (30.1%); Class I2, 9 (3.4%); Class I3, 73 (27.3%); Class I4, 16 (6.0%); and Class I5, 87 (32.6%). The leading causes of death were respiratory diseases, 92 (34.5%); circulatory diseases, 61 (22.8%); and senility, 50 (18.7%) (Table 3).

The characteristics of each cluster, listed in order of number of persons, were as follows. A typical examples of Class I5 was "those exhibiting decreased social activity and depression, and receiving care certification" {decreased social participation [yes, 61 (70.1%) and no, 0 (0.0%), p < 0.001,V = 0.73], depression [yes, 47 (54.0%) and no, 0 (0.0%), p < 0.001, V = 0.86], receiving care certification [yes, 67 (77.0%) and no, 12 (13.8%), p = 0.002, V = 0.25], disability [yes, 0 (0.0%) and no, 78 (89.7%), p < 0.001, V = 0.34], and the number of days from the earthquake to death (174.6 ± 144.4, p < 0.001, η^2^ = 0.11)}. For Class I1, a typical example was “those who moved out of the prefecture, were not depressed, and were receiving care certification” {depression [yes, 7 (8.5%) and no, 56 (68.3%), p < 0.001, V = 0.86], receiving care certification [yes, 61 (74.4%) and no, 13 (15.9%), p = 0.002, V = 0.25], moving location [in-prefecture, 20 (24.4%) and outside, 59 (62.0%), p < 0.001, V = 0.35], disability [yes, 13 (15.9%) and no, 63 (76.8%), p < 0.001, V = 0.34], and the number of days from the earthquake to death (90.2 ± 95.0, p < 0.001, η^2^ = 0.11)}. Class I3 comprised "those who stayed in the prefecture, were not depressed or disabled, and were hospitalized at the time of the disaster" {moving location [in-prefecture, 42 (57.5%) and out-of-prefecture, 28 (38.4%), p < 0.001,V = 0.35], status at the time of the disaster [in hospitals, 56 (76.7%) and in institutions, 17 (23.3%), p < 0.001,V = 0.29], receiving care certification [yes, 37 (50.7%) and no, 24 (32.9%), p = 0.002,V = 0.25], disability [yes, 0 (0.0%) and no, 59 (80.8%), p < 0.001,V = 0.34], and the number of days from the earthquake to death (94.5 ± 92.5, p < 0.001, η^2^ = 0.11)}. A typical example of Class I4 was "those with disability and receiving care certification" {receiving care certification [yes, 12 (75.0%) and no, 4 (25.0%)], disability [yes, 16 (100.0%) and no, 0 (0.0%)], and the number of days from the earthquake to death (666.7 ± 500.2)}. Class I2 comprised "those who did not evacuate after the disaster" {evacuation [yes, 0 (0.0%) and no, 9 (100.0%)], receiving care certification [yes, 4 (44.4%) and no, 5 (55.6%)], disability [yes, 0 (0.0%) and no, 9 (100.0%)], and number of days from the earthquake to death (40.1 ± 65.1)}.

Factors that largely distinguished the characteristics of each group were the presence or absence of a disability, receiving care certification, evacuation, and depression (Fig. 4).

## Discussion

In this study, the average age of those identified as disaster-related deaths was 82.7 ± 11.9 years, which is consistent with previous reports that these deaths tend to occur in people in their 70 s and 80 s, with no significant sex bias^7,10,40^. Among people who were at home at the time of the disaster, only three of the seven groups received nursing care, whereas among those in a hospital or facility, only one of the five groups did not receive nursing care. This analysis is significant, as it helped extract a population that did not receive nursing care from a population with many care recipients and vice versa.

This study showed that disaster-related deaths in a radiation disaster can be divided into seven groups for those who were at home at the time of the disaster and into five groups for those who were in a hospital or at a facility at the time of the disaster. Clear differences were evident in the groups' causes of death. The people at home had more deaths from circulatory diseases and malignant neoplasms, which accounted for approximately half of all deaths. While those in a hospital or facility had more deaths due to respiratory diseases and senility, which also accounted for one-third of all deaths. The two groups also differed in terms of the diseases to which they were prone.

However, the two groups also shared some similarities. Regardless of the location of the disaster, people in the groups that had a longer period between the disaster and their death experienced depressive feelings. Most of the individuals belonging to the people at home and in a hospital or facility exhibited depressive feelings after an average of 100 and 170 days, respectively, suggesting that they may have been more likely to become depressed 3 months following the disaster. This is possibly because long-term evacuation prolongs the effects of environmental changes, which consequently increases the psychological impact on the survivors, thus raising the risk of death. This is consistent with previous studies which reported that long-term depressive feelings after disasters are more likely to lead to death^25,26,41,42^.

Notably, that the time to death in clusters I4 and H5, which included people with disabilities, was approximately 610–670 days, which was longer in comparison to that in other clusters, suggesting that the characteristics of disaster-related deaths among people with disabilities may differ from those of other groups. The reason for this is thought to be that disaster victims weaken over the long term due to prolonged evacuation and changes in post-disaster medical services that gradually affect their lives. Disabilities that did not have a significant impact on them before the disaster gradually begins to affect their lives. Previous studies have reported that people with disabilities take longer to recover after disasters^8,43^ and there have been cases of people dying from long-term effects of disasters^37^. These findings are consistent with the present study results, which showed that people with disabilities succumb to long-term effects. Interview research to clarify how people with disabilities are affected and weakened is warranted in the future.

People living in institutions or hospitals at the time of the disaster were divided into three groups according to the duration of time before their death. Individuals belonging to one group died within 2 months, another within 3–6 months, and the other within 6 months due to the acute, subacute, and chronic effects after the earthquake, respectively. Previous similar studies have shown that people in facilities die within one to two months after a disaster, primarily due to evacuation^44^. However, in this study, we found that some people died before evacuation, for example class I^2^, and others died after six months, such as class I4 or I5, which had not been found in previous studies. Future studies are required to accumulate more research evidence regarding such groups.

This study revealed that people without receiving and not receiving nursing care who were living at home at the time of the disaster and had depressive tendencies could be divided into two main categories: those with reduced social activity and communication with others (such as class H5) and those without these characteristics (such as class H3). In the first category, it is considered that the environmental factors due to prolonged evacuation resulted in reduced social activity and caused depressive tendencies. However, in the second group, it is contemplated that these characteristics were not related to the disaster, but occurred because the person originally showed depressive tendencies, such as mental illness and cancer. Previous studies^45–48^ have suggested that people with depression were affected by changes in the social environment after the earthquake. However, some people who have depression without such effects, which seems to be a new finding.

An important finding is that while some debilitated people who originally had serious illnesses died within three months of the disaster, others lingered beyond three months and developed depression. Moreover, these long-term survivors not only included originally healthy individuals, but also those with disabilities and pre-existing medical conditions. These facts suggest that post-disaster support for disaster victims may require not only uniform support, but also group-specific continuous support. In addition, mental health care was required 3 months after the disaster. These are important findings on chronic disaster care for future disaster medicine.

## Limitations

However, this study had a few limitations which need consideration. First, the classifications are used to observe group tendencies and do not consider every individual’s behavior. This aspect should be considered when conducting qualitative surveys in the future to determine individual characteristics. Furthermore, a more detailed analysis with the population stratified by various demographic factors, such as age and sex, is necessary. Second, data were registered based on applications from family members of those who died in disaster-related deaths, which were classified by the authors. This may have resulted in some bias because some parts were not based on objective indicators. However, the judgments by multiple raters would have reduced this bias. Third, there was a large amount of missing data regarding questions such as motivation to engage in social activities and depressive tendencies, with sleep disorders at the top of the list. These were complemented by the multiple assignment method, which has limited accuracy. However, when a similar analysis was conducted using only data that did not include missing values, the participants were classified into similar clusters, suggesting that the multiple assignment method is a reasonable complement.

## Conclusion

Radiation disaster-related deaths can be divided into seven categories for those who were at home at the time of the disaster and into five categories for those who were in hospitals or facilities at the time of the disaster. These clusters can be classified based on “residential status at the time of the disaster,” “receiving long-term care certification,” “presence of disabilities,” “presence of depression,” and “experience of evacuation.” Moreover, these clusters included “people who did not evacuate after the disaster and stayed at home,” “people with disabilities,” “people receiving care,” and “people with depressive feelings.” Some of the individuals belonging to these categories originally had serious illnesses and died within three months of the disaster, while others survived until after three months and began to experience depressive feelings. In addition, each category had distinct diseases that were likely to be the cause of death, such as “the group with more deaths from malignant neoplasms,” “the group with more deaths from respiratory diseases,” and “the group with more deaths from debilitating diseases”. The prolonged group not only included those who were originally healthy, but also those with disabilities and pre-existing medical conditions. These findings suggest that assisting disaster victims not only require uniform support, but also individual support based on the group and category they belong to.

### Supplementary Information

Supplementary Table 1.

## Data Availability

All data generated or analyzed during this study are included in this published article.
