# Publisher Correction: Categorization of disaster-related deaths in Minamisoma city after the Fukushima nuclear disaster using clustering analysis

**DOI:** 10.1038/s41598-024-54746-x

**Published:** 2024-02-20

**Authors:** Hiroki Yoshimura, Toyoaki Sawano, Michio Murakami, Yuna Uchi, Moe Kawashima, Kemmei Kitazawa, Saori Nonaka, Naomi Ito, Hiroaki Saito, Toshiki Abe, Nobuaki Moriyama, Mamoru Sakakibara, Kazuko Yagiuchi, Mako Otsuki, Arinobu Hori, Akihiko Ozaki, Chika Yamamoto, Tianchen Zhao, Taiga Uchiyama, Tomoyoshi Oikawa, Shinichi Niwa, Masaharu Tsubokura

**Affiliations:** 1 Department of Radiation Health Management, Fukushima Medical University School of Medicine, Fukushima, Japan; 2 School of Medicine, Hiroshima University, Hiroshima, Japan; 3 Department of Surgery, Jyoban Hospital of Tokiwa Foundation, Iwaki, Japan; 4 Research Center for Community Health, Minamisoma Municipal General Hospital, Fukushima, Japan; 5 Department of Health Risk Communication, Fukushima Medical University School of Medicine, Fukushima, Japan; 6 Present Address: Center for Infectious Disease Education and Research, Osaka University, Suita, Japan; 7 Department of Internal Medicine, Soma Central Hospital, Fukushima, Japan; 8 Department of Public Health, Fukushima Medical University School of Medicine, Fukushima, Japan; 9 Reinstatement Support Center for Nurses, Incorporated Foundation of Tokiwa-Kai, Iwaki, Japan; 10 St. Olive Nursing Home, Shirakawa, Japan; 11 Department of Nursing, Fukushima Medical University Hospital, Fukushima, Japan; 12 Department of Psychiatry, Hori Mental Clinic, Minamisoma, Japan; 13 Department of Breast and Thyroid Surgery, Jyoban Hospital of Tokiwa Foundation, Iwaki, Japan; 14 Department of Neurosurgery, Minamisoma Municipal General Hospital, Minamisoma, Japan; 15 Department of Psychiatry, Aizu Medical Center, Fukushima Medical University, Aizuwakamatsu, Japan

Correction to: *Scientific Reports* 10.1038/s41598-024-53165-2, published online 05 February 2024

In the original version of this Article Masaharu Tsubokura was incorrectly affiliated with ‘Department of Psychiatry, Aizu Medical Center, Fukushima Medical University, Aizuwakamatsu, Japan’. Their correct affiliations are listed below.

Department of Radiation Health Management, Fukushima Medical University School of Medicine, Fukushima, Japan.

Research Center for Community Health, Minamisoma Municipal General Hospital, Fukushima, Japan.

The original Article has been corrected.

