# Supplementary material for: Categorization of disaster-related deaths in Minamisoma city after the Fukushima nuclear disaster using clustering analysis
Source: Sci Rep. 2024 Feb 5;14:2946. doi: 10.1038/s41598-024-53165-2 (PMC10844307; doi:10.1038/s41598-024-53165-2)
Supplement: Supplementary file 1 — Supplementary Table 1. [file 41598_2024_53165_MOESM1_ESM.docx]

Supplemental Table1: Comparison of background information between those who were at home and those who were in hospitals/facilities at the time of the disaster.

| Variable | Category | Home | Hospital/Facility | P value* |
| --- | --- | --- | --- | --- |
| Sex | female | 101 | 151 | <0.001 |
|  | male | 151 | 116 |  |
| Evacuation | yes | 235 | 258 | 0.12 |
|  | no | 17 | 9 |  |
| Situation when moving | Accompanied | 218 | 75 | <0.001 |
|  | Unaccompanied | 14 | 175 |  |
| Moving Location | Within the prefecture | 83 | 74 | 0.15 |
|  | Outside the prefecture | 151 | 182 |  |
| Roommate | Yes | 208 | 112 | <0.001 |
|  | No | 40 | 153 |  |
| Evacuation housing | Temporary housing | 6 | 0 | <0.001 |
|  | Temporary facility | 2 | 0 |  |
|  | Rental housing | 52 | 5 |  |
|  | Facility | 57 | 110 |  |
|  | Hospital | 22 | 119 |  |
|  | Home | 19 | 6 |  |
|  | Relative's house | 67 | 17 |  |
| Household | Single | 19 | 36 | <0.001 |
|  | Facility | 13 | 83 |  |
|  | One | 92 | 43 |  |
|  | Two | 82 | 67 |  |
|  | Three | 41 | 33 |  |
|  | Four | 2 | 4 |  |
| Disabilities | yes | 26 | 29 | 0.80 |
|  | no | 210 | 209 |  |
| Nursing care | yes | 90 | 181 | <0.001 |
|  | no | 143 | 58 |  |
| Increased drinking | yes | 4 | 1 | 0.20 |
|  | no | 168 | 239 |  |
| Increased insomnia | yes | 79 | 26 | <0.001 |
|  | no | 28 | 68 |  |
| Depression | yes | 155 | 88 | <0.001 |
|  | no | 18 | 62 |  |
| Worsening of dementia | yes | 51 | 49 | 0.85 |
|  | no | 88 | 78 |  |
| Decreased social participation | yes | 177 | 121 | <0.001 |
|  | no | 35 | 68 |  |
| Decreased communication | yes | 109 | 98 | 0.71 |
|  | no | 68 | 68 |  |
| Place of death | Facility | 6 | 30 | <0.001 |
|  | Hospital | 207 | 228 |  |
|  | Home | 26 | 4 |  |
|  | Refuge | 7 | 0 |  |
| Residence Classification(3/11) | over30km | 42 | 77 | 0.001 |
|  | under20km | 210 | 190 |  |
| Ward(3/11) | Haramachi | 146 | 175 | 0.002 |
|  | Odaka | 83 | 50 |  |
|  | Kashima | 23 | 33 |  |
| Direct cause of death | A00-B99 | 5 | 3 | <0.001 |
|  | C00-D48 | 59 | 19 |  |
|  | D50-D89 | 2 | 2 |  |
|  | E00-E90 | 1 | 0 |  |
|  | G00-G99 | 0 | 3 |  |
|  | I00-I99 | 62 | 61 |  |
|  | J00-J99 | 51 | 92 |  |
|  | K00-K93 | 7 | 8 |  |
|  | M00-M99 | 0 | 2 |  |
|  | N00-N99 | 9 | 16 |  |
|  | R00-R99 | 37 | 50 |  |
|  | S00-T98 | 2 | 1 |  |
|  | V01-Y98 | 17 | 2 |  |
| Age at death |  | 80.7±13.6 | 84.6±9.75 | <0.001 |
| Number of evacuations |  | 2.44±1.69 | 1.64±1.17 | <0.001 |
| Number of Movements |  | 3.73±2.51 | 2.38±1.83 | <0.001 |
| Days since the earthquake |  | 314.2±371.0 | 151.7±211.3 | <0.001 |

* Chi-square test for categorical variables, one-way ANOVA otherwise.
